# Ethyl 5-oxo-2,3-diphenyl­cyclo­pentane-1-carboxyl­ate

**DOI:** 10.1107/S1600536810014728

**Published:** 2010-04-28

**Authors:** Claude N. Lamb, Zerihun Assefa, Richard E. Sykora

**Affiliations:** a North Carolina A&T State University, Department of Chemistry, Greensboro, NC 27411, USA; b University of South Alabama, Department of Chemistry, Mobile, AL 36688-0002, USA

## Abstract

The title compound, C_20_H_20_O_3_, was prepared by an acyl­oin-type condensation reaction in the presence of sodium sand and dry ether using ethyl cinnamate as the starting material. The C—O bond lengths for the carbonyl groups are 1.191 (2) and 1.198 (2) Å, while the C—O bond in the ester group is 1.335 (2) Å. The C—C bond lengths in the phenyl groups average 1.375 Å, while the C—C bonds in the cyclo­penta­none ring average 1.525 Å, indicating single C—C bonds in the latter.

## Related literature

For the first synthesis of the title compound, see: Totton *et al.* (1965 ▶). For general methods of β-keto ester preparation, see: March (1985 ▶); Shiosaki *et al.*(1981 ▶); Matsumoto *et al.* (1973 ▶). For acyl­oin-type condensation reactions of α, β unsaturated esters, see: Totton *et al.* (1961 ▶, 1965 ▶, 1967 ▶); Singh & Totton (1981 ▶). The mechanism of this condensation reaction was first suggested by Weidlich (1938 ▶) and confirmed by the successful synthesis of several adducts.
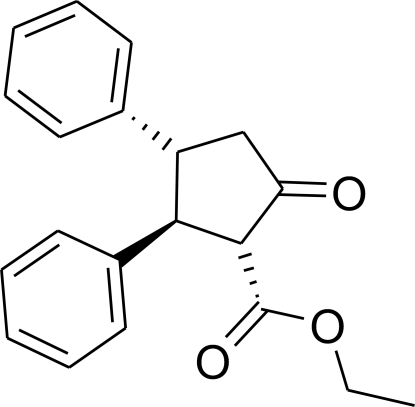


## Experimental

### 

#### Crystal data

C_20_H_20_O_3_
*M* *_r_* = 308.36
Monoclinic, 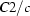

*a* = 27.4961 (13) Å
*b* = 7.4008 (2) Å
*c* = 18.7063 (10) Å
β = 115.389 (6)°
*V* = 3439.0 (3) Å^3^
*Z* = 8
Mo *K*α radiation
μ = 0.08 mm^−1^
*T* = 295 K
0.14 × 0.14 × 0.08 mm

#### Data collection

Oxford Diffraction Xcalibur Eos diffractometer
Absorption correction: analytical [*CrysAlis PRO* (Oxford Diffraction, 2009 ▶) and Clark & Reid (1995 ▶)] *T*_min_ = 0.990, *T*_max_ = 0.994
6900 measured reflections
3025 independent reflections
1509 reflections with *I* > 2σ(*I*)
*R*_int_ = 0.018

#### Refinement

*R*[*F* ^2^ > 2σ(*F* ^2^)] = 0.040
*wR*(*F* ^2^) = 0.108
*S* = 0.83
3025 reflections
210 parameters
H-atom parameters constrained
Δρ_max_ = 0.25 e Å^−3^
Δρ_min_ = −0.13 e Å^−3^

### 

Data collection: *CrysAlis PRO* (Oxford Diffraction, 2009 ▶); cell refinement: *CrysAlis PRO*; data reduction: *CrysAlis PRO*; program(s) used to solve structure: *SHELXS97* (Sheldrick, 2008 ▶); program(s) used to refine structure: *SHELXL97* (Sheldrick, 2008 ▶); molecular graphics: *SHELXTL* (Sheldrick, 2008 ▶); software used to prepare material for publication: *publCIF* (Westrip, 2010 ▶).

## Supplementary Material

Crystal structure: contains datablocks I, global. DOI: 10.1107/S1600536810014728/hg2655sup1.cif

Structure factors: contains datablocks I. DOI: 10.1107/S1600536810014728/hg2655Isup2.hkl

Additional supplementary materials:  crystallographic information; 3D view; checkCIF report

## Figures and Tables

**Fig. 1.**
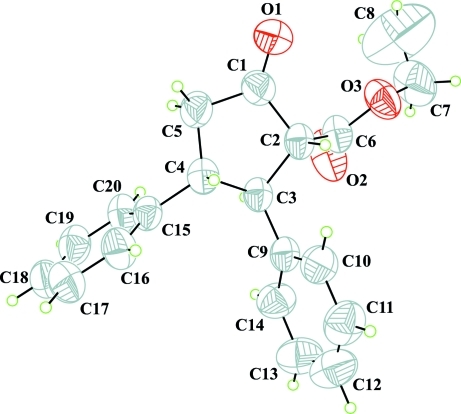
The molecular structure of I, with the atom-numbering scheme. Displacement ellipsoids for non-hydrogen atoms are drawn at the 50% probability level.

## supplementary crystallographic information

### Comment

β-keto esters are a class of potentially useful synthetic intermediates in the preparation of some physiologically active compounds. The medicinal values of this class of compounds have been demonstrated as antitumor, antianxiety, and antihypertension agents. General methods of β-keto ester preparation have been described in several publications including by March (1985), Shiosaki *et al.*(1981), and Matsumoto *et al.* (1973). Acyloin-type condensation reactions of α, β unsaturated esters have also been demonstrated in several publications of Totton *et al.* (1961), (1965), (1967), and Singh & Totton (1981). The mechanism of this condensation reaction was first suggested by Weidlich (1938) and confirmed by the successful synthesis of several adducts. Synthesis of the title compound was first performed by Totton *et al.* (1965). However, the compound has not previously been characterized by X-ray diffraction and therefore these studies were undertaken in order to elucidate details of the molecular structure. The title compound, C_20_H_20_O_3_, contains three chiral centers. These correspond to atom sites C2, C3, and C4 and contain R, S, and R configurations, respectively. The C—O bond lengths for the two carbonyl groups are 1.191 (2) and 1.198 (2) Å with the ring carbonyl having the slightly longer distance. The C—O bond in the ester group is quite a bit longer than the carbonyl distances, as expected, at 1.335 (2) Å. The aromatic C—C bond lengths in the phenyl groups are not extraordinary and average to 1.375 Å, while the C—C bonds in the cyclopentanone ring have an average distance of 1.525 Å indicative of the single bond nature. The molecular nature of the compound is preserved in the solid state. No significant interactions, e.g. H-bonding interactions, etc., are observed in the structure.

### Experimental

The synthesis of the (1*R*,2S,3*R*)-ethyl 5-oxo-2,3-diphenylcyclopentanecarboxylate product was accomplished by modifi-cation of the prior procedure used by Totton (1961). Into a 1 L three necked round bottom flask fitted with a reflux condenser containing a CaCl_2_ drying tube was added 400 ml dry ether and 13 g of freshly prepared sodium sand, 50 g of ethyl cinnamate (287.1 mmol) was then added dropwise over a period of two hours. A series of color changes were observed where the initial light orange color changed to deep orange and finally to reddish brown. The mixture was stirred and refluxed overnight and cooled in an ice bath. While stirring, 70 ml of a 35% sulfuric acid was added carefully through an addition funnel. The reaction turned to yellow-orange color. The mixture was transferred to a large separatory funnel and the layers separated. The aqueous layer was then extracted with two – 75 ml portions of ether and combined to the original ether layer and which was then washed with four – 50 ml portions of a 20 % sodium carbonate solution and 100 ml water. The ether solution was dried over 50 g of anhydrous sodium sulfate, filtered by gravity, and the solvent removed with a rotatory evaporator. The sticky residue was dissolved in 200 ml of 95% ethanol and left for 1 hr at room temperature and kept in freezer overnight. The product was recrystallized several times from a 95% ethanol/water mixture. Yield was 10 %.

The compound is soluble in a number of organic solvents including diethyl ether, dichloromethane, methanol, ethanol etc, but found insoluble in hexane and hence single crystals for X-ray measurements were grown from an ether/hexane mixture.

The product was characterized using several spectroscopic techniques in addition to the X-ray analysis. The melting point was sharp (97-99 °C). 1*H*-NMR (DMSO): 7.2 m (10 H), 4.1 m (2 H), 3.9 t (1 H), 3.5 m (2 H), 2.98 q (1 H), 2.68 m (1 H), 1.2 m (3 H). IR spectrum: 2960-3057 cm^-1^ for C—H symmetric stretch; 1728, 1752 cm^-1^ for the C=O group and at ~1130 cm^-1^ for the C—O ether linkage. The 700-756 cm^-1^ region corresponds to the aromatic ring. The mass spectrum indicates a loss of the carbethoxy fragment from the molecular ion ( MW = 308), as represented by the peak with m/e of 236. Other stable fragment ions are represented by peaks at m/e of 178, 105, 104, and 77 indicating loss of various components of the material.

The compound shows a bright blue unstructured emission covering the 400-600 nm s pectral region at room temperature with the emission band maximizing at 460 nm. The excitation spectrum displays two broad bands at 310 nm and 405 nm. At liquid N_2_ temperature well defined bands are observed at 440 and 480 nm with a shoulder at 520 nm. The excitation band at liquid N_2_ temperature is also broad, centering at 380 nm. The overall emission spectrum is unaffected upon changing the excitation wavelength.

### Refinement

H-atoms were placed in calculated positions and allowed to ride during subsequent refinement, with *U*_iso_(H) = 1.2*U*_eq_(C) and C—H distances of 0.93 Å for the aromatic H atoms, with *U*_iso_(H) = 1.2*U*_eq_(C) and C—H distances of 0.98 Å for tertiary H atoms, with *U*_iso_(H) = 1.2*U*_eq_(C) and C—H distances of 0.97 Å for secondary H atoms, and with *U*_iso_(H) = 1.5*U*_eq_(C) and C—H distances of 0.96 Å for methyl H atoms. The terminal methyl group corresponding to C8 has a relatively large thermal ellipsoid corresponding to a high degree of thermal motion.

### Crystal data

**Table:**

C_20_H_20_O_3_	*F*(000) = 1312
*M**_r_* = 308.36	*D*_x_ = 1.191 Mg m^−^^3^
Monoclinic, *C*2/*c*	Mo *K*α radiation, λ = 0.71073 Å
Hall symbol: -C 2yc	Cell parameters from 2093 reflections
*a* = 27.4961 (13) Å	θ = 3.0–25.3°
*b* = 7.4008 (2) Å	µ = 0.08 mm^−^^1^
*c* = 18.7063 (10) Å	*T* = 295 K
β = 115.389 (6)°	Plate, colorless
*V* = 3439.0 (3) Å^3^	0.14 × 0.14 × 0.08 mm
*Z* = 8	

### Data collection

**Table:**

Oxford Diffraction Xcalibur Eos diffractometer	3025 independent reflections
Radiation source: fine-focus sealed tube	1509 reflections with *I* > 2σ(*I*)
graphite	*R*_int_ = 0.018
Detector resolution: 16.0514 pixels mm^-1^	θ_max_ = 25.0°, θ_min_ = 3.0°
ω scans	*h* = −32→31
Absorption correction: analytical [*CrysAlis PRO* (Oxford Diffraction, 2009) and Clark & Reid (1995)]	*k* = −8→8
*T*_min_ = 0.990, *T*_max_ = 0.994	*l* = −22→22
6900 measured reflections	

### Refinement

**Table:**

Refinement on *F*^2^	Secondary atom site location: difference Fourier map
Least-squares matrix: full	Hydrogen site location: inferred from neighbouring sites
*R*[*F*^2^ > 2σ(*F*^2^)] = 0.040	H-atom parameters constrained
*wR*(*F*^2^) = 0.108	*w* = 1/[σ^2^(*F*_o_^2^) + (0.0618*P*)^2^] where *P* = (*F*_o_^2^ + 2*F*_c_^2^)/3
*S* = 0.83	(Δ/σ)_max_ < 0.001
3025 reflections	Δρ_max_ = 0.25 e Å^−^^3^
210 parameters	Δρ_min_ = −0.13 e Å^−^^3^
0 restraints	Extinction correction: *SHELXL97* (Sheldrick, 2008), Fc^*^=kFc[1+0.001xFc^2^λ^3^/sin(2θ)]^-1/4^
Primary atom site location: structure-invariant direct methods	Extinction coefficient: 0.0016 (4)

### Special details

**Table:**

Geometry. All esds (except the esd in the dihedral angle between two l.s. planes) are estimated using the full covariance matrix. The cell esds are taken into account individually in the estimation of esds in distances, angles and torsion angles; correlations between esds in cell parameters are only used when they are defined by crystal symmetry. An approximate (isotropic) treatment of cell esds is used for estimating esds involving l.s. planes.
Refinement. Refinement of *F*^2^ against ALL reflections. The weighted *R*-factor wR and goodness of fit *S* are based on *F*^2^, conventional *R*-factors *R* are based on *F*, with *F* set to zero for negative *F*^2^. The threshold expression of *F*^2^ > 2σ(*F*^2^) is used only for calculating *R*-factors(gt) etc. and is not relevant to the choice of reflections for refinement. *R*-factors based on *F*^2^ are statistically about twice as large as those based on *F*, and *R*- factors based on ALL data will be even larger.

### Fractional atomic coordinates and isotropic or equivalent isotropic displacement parameters (Å^2^)

**Table:**

	*x*	*y*	*z*	*U*_iso_*/*U*_eq_	
C1	0.07043 (8)	0.5517 (3)	0.12865 (12)	0.0674 (6)	
C2	0.07109 (7)	0.4093 (2)	0.07003 (11)	0.0555 (5)	
H2	0.0459	0.4470	0.0167	0.067*	
C3	0.12801 (6)	0.4166 (2)	0.07539 (10)	0.0512 (5)	
H3	0.1511	0.3419	0.1205	0.061*	
C4	0.14455 (7)	0.6168 (2)	0.09701 (11)	0.0556 (5)	
H4	0.1260	0.6894	0.0491	0.067*	
C5	0.12035 (7)	0.6635 (2)	0.15433 (12)	0.0679 (6)	
H5A	0.1453	0.6342	0.2082	0.082*	
H5B	0.1117	0.7912	0.1515	0.082*	
C6	0.05346 (8)	0.2295 (3)	0.08650 (12)	0.0648 (6)	
C7	−0.02207 (10)	0.0601 (3)	0.0812 (2)	0.1136 (10)	
H7A	−0.0551	0.0236	0.0369	0.136*	
H7B	0.0034	−0.0389	0.0935	0.136*	
C8	−0.0323 (2)	0.0926 (5)	0.1442 (3)	0.248 (3)	
H8A	0.0011	0.1017	0.1909	0.371*	
H8B	−0.0533	−0.0047	0.1503	0.371*	
H8C	−0.0519	0.2038	0.1364	0.371*	
C9	0.13429 (7)	0.3507 (2)	0.00345 (11)	0.0555 (5)	
C10	0.10049 (8)	0.4082 (3)	−0.07179 (12)	0.0702 (6)	
H10	0.0728	0.4883	−0.0787	0.084*	
C11	0.10716 (10)	0.3488 (3)	−0.13712 (14)	0.0880 (7)	
H11	0.0839	0.3884	−0.1874	0.106*	
C12	0.14780 (12)	0.2323 (3)	−0.12798 (17)	0.0965 (8)	
H12	0.1523	0.1922	−0.1719	0.116*	
C13	0.18164 (11)	0.1751 (3)	−0.05443 (18)	0.0934 (7)	
H13	0.2095	0.0963	−0.0481	0.112*	
C14	0.17513 (8)	0.2328 (3)	0.01133 (14)	0.0759 (6)	
H14	0.1985	0.1916	0.0613	0.091*	
C15	0.20420 (7)	0.6534 (2)	0.12792 (11)	0.0533 (5)	
C16	0.22364 (8)	0.7672 (3)	0.08826 (13)	0.0715 (6)	
H16	0.1998	0.8202	0.0412	0.086*	
C17	0.27797 (10)	0.8043 (3)	0.11702 (16)	0.0886 (7)	
H17	0.2904	0.8824	0.0895	0.106*	
C18	0.31355 (9)	0.7261 (3)	0.18599 (16)	0.0832 (7)	
H18	0.3502	0.7507	0.2055	0.100*	
C19	0.29501 (8)	0.6119 (3)	0.22596 (13)	0.0757 (6)	
H19	0.3191	0.5579	0.2726	0.091*	
C20	0.24114 (8)	0.5764 (2)	0.19776 (12)	0.0655 (6)	
H20	0.2290	0.4993	0.2259	0.079*	
O1	0.03553 (6)	0.5692 (2)	0.15029 (11)	0.1100 (6)	
O2	0.08328 (6)	0.10985 (18)	0.12097 (10)	0.1022 (6)	
O3	−0.00002 (5)	0.21897 (16)	0.05868 (9)	0.0815 (5)	

### Atomic displacement parameters (Å^2^)

**Table:**

	*U*^11^	*U*^22^	*U*^33^	*U*^12^	*U*^13^	*U*^23^
C1	0.0573 (13)	0.0655 (12)	0.0769 (15)	0.0007 (11)	0.0264 (11)	−0.0052 (11)
C2	0.0520 (11)	0.0482 (10)	0.0563 (11)	−0.0013 (9)	0.0139 (9)	0.0025 (9)
C3	0.0451 (10)	0.0444 (10)	0.0538 (11)	0.0029 (8)	0.0114 (8)	0.0026 (8)
C4	0.0514 (11)	0.0451 (10)	0.0611 (12)	0.0034 (9)	0.0154 (9)	0.0030 (9)
C5	0.0612 (12)	0.0552 (11)	0.0809 (15)	−0.0014 (10)	0.0243 (11)	−0.0128 (10)
C6	0.0547 (13)	0.0601 (13)	0.0678 (14)	−0.0045 (11)	0.0150 (10)	0.0008 (11)
C7	0.112 (2)	0.0680 (14)	0.178 (3)	−0.0309 (14)	0.078 (2)	−0.0002 (17)
C8	0.460 (8)	0.151 (3)	0.270 (6)	−0.108 (4)	0.289 (6)	−0.023 (3)
C9	0.0551 (11)	0.0477 (10)	0.0594 (13)	−0.0071 (9)	0.0205 (10)	−0.0030 (9)
C10	0.0690 (13)	0.0769 (13)	0.0612 (14)	0.0009 (11)	0.0244 (11)	0.0017 (11)
C11	0.1040 (18)	0.0914 (16)	0.0651 (16)	−0.0094 (15)	0.0329 (13)	0.0038 (13)
C12	0.136 (2)	0.0823 (16)	0.094 (2)	−0.0037 (17)	0.0707 (19)	−0.0059 (15)
C13	0.1072 (19)	0.0830 (15)	0.106 (2)	0.0188 (14)	0.0606 (17)	−0.0007 (16)
C14	0.0782 (14)	0.0709 (13)	0.0797 (16)	0.0135 (12)	0.0350 (12)	−0.0014 (12)
C15	0.0521 (11)	0.0426 (10)	0.0593 (12)	−0.0023 (9)	0.0182 (10)	−0.0046 (9)
C16	0.0681 (14)	0.0692 (12)	0.0725 (15)	−0.0103 (11)	0.0256 (11)	0.0051 (11)
C17	0.0874 (18)	0.0887 (16)	0.100 (2)	−0.0273 (14)	0.0499 (15)	−0.0109 (15)
C18	0.0583 (14)	0.0838 (15)	0.104 (2)	−0.0127 (12)	0.0314 (15)	−0.0300 (15)
C19	0.0606 (14)	0.0659 (12)	0.0816 (15)	0.0022 (11)	0.0124 (12)	−0.0103 (12)
C20	0.0557 (12)	0.0570 (11)	0.0691 (14)	−0.0032 (10)	0.0126 (10)	0.0010 (10)
O1	0.0867 (12)	0.1232 (13)	0.1423 (16)	−0.0258 (10)	0.0702 (11)	−0.0533 (11)
O2	0.0727 (10)	0.0656 (9)	0.1337 (15)	−0.0009 (8)	0.0113 (9)	0.0322 (9)
O3	0.0626 (9)	0.0668 (8)	0.1157 (13)	−0.0089 (7)	0.0388 (8)	0.0037 (8)

### Geometric parameters (Å, °)

**Table:**

C1—O1	1.198 (2)	C9—C14	1.379 (2)
C1—C5	1.495 (2)	C9—C10	1.380 (3)
C1—C2	1.527 (3)	C10—C11	1.383 (3)
C2—C6	1.493 (2)	C10—H10	0.9300
C2—C3	1.526 (2)	C11—C12	1.363 (3)
C2—H2	0.9800	C11—H11	0.9300
C3—C9	1.509 (2)	C12—C13	1.356 (3)
C3—C4	1.552 (2)	C12—H12	0.9300
C3—H3	0.9800	C13—C14	1.384 (3)
C4—C15	1.511 (2)	C13—H13	0.9300
C4—C5	1.525 (3)	C14—H14	0.9300
C4—H4	0.9800	C15—C16	1.373 (3)
C5—H5A	0.9700	C15—C20	1.388 (2)
C5—H5B	0.9700	C16—C17	1.381 (3)
C6—O2	1.191 (2)	C16—H16	0.9300
C6—O3	1.335 (2)	C17—C18	1.369 (3)
C7—C8	1.344 (4)	C17—H17	0.9300
C7—O3	1.465 (2)	C18—C19	1.364 (3)
C7—H7A	0.9700	C18—H18	0.9300
C7—H7B	0.9700	C19—C20	1.368 (3)
C8—H8A	0.9600	C19—H19	0.9300
C8—H8B	0.9600	C20—H20	0.9300
C8—H8C	0.9600		
			
O1—C1—C5	126.14 (19)	H8A—C8—H8C	109.5
O1—C1—C2	125.13 (18)	H8B—C8—H8C	109.5
C5—C1—C2	108.73 (17)	C14—C9—C10	117.8 (2)
C6—C2—C3	115.64 (15)	C14—C9—C3	120.56 (17)
C6—C2—C1	111.23 (16)	C10—C9—C3	121.67 (17)
C3—C2—C1	104.83 (14)	C9—C10—C11	121.1 (2)
C6—C2—H2	108.3	C9—C10—H10	119.5
C3—C2—H2	108.3	C11—C10—H10	119.5
C1—C2—H2	108.3	C12—C11—C10	120.2 (2)
C9—C3—C2	115.86 (14)	C12—C11—H11	119.9
C9—C3—C4	114.09 (15)	C10—C11—H11	119.9
C2—C3—C4	103.23 (13)	C13—C12—C11	119.6 (2)
C9—C3—H3	107.7	C13—C12—H12	120.2
C2—C3—H3	107.7	C11—C12—H12	120.2
C4—C3—H3	107.7	C12—C13—C14	120.8 (2)
C15—C4—C5	114.81 (15)	C12—C13—H13	119.6
C15—C4—C3	114.83 (13)	C14—C13—H13	119.6
C5—C4—C3	103.44 (14)	C9—C14—C13	120.6 (2)
C15—C4—H4	107.8	C9—C14—H14	119.7
C5—C4—H4	107.8	C13—C14—H14	119.7
C3—C4—H4	107.8	C16—C15—C20	117.59 (17)
C1—C5—C4	105.42 (16)	C16—C15—C4	120.89 (16)
C1—C5—H5A	110.7	C20—C15—C4	121.51 (17)
C4—C5—H5A	110.7	C15—C16—C17	121.2 (2)
C1—C5—H5B	110.7	C15—C16—H16	119.4
C4—C5—H5B	110.7	C17—C16—H16	119.4
H5A—C5—H5B	108.8	C18—C17—C16	120.0 (2)
O2—C6—O3	123.72 (18)	C18—C17—H17	120.0
O2—C6—C2	124.42 (17)	C16—C17—H17	120.0
O3—C6—C2	111.86 (17)	C19—C18—C17	119.6 (2)
C8—C7—O3	112.1 (2)	C19—C18—H18	120.2
C8—C7—H7A	109.2	C17—C18—H18	120.2
O3—C7—H7A	109.2	C18—C19—C20	120.3 (2)
C8—C7—H7B	109.2	C18—C19—H19	119.8
O3—C7—H7B	109.2	C20—C19—H19	119.8
H7A—C7—H7B	107.9	C19—C20—C15	121.2 (2)
C7—C8—H8A	109.5	C19—C20—H20	119.4
C7—C8—H8B	109.5	C15—C20—H20	119.4
H8A—C8—H8B	109.5	C6—O3—C7	117.20 (17)
C7—C8—H8C	109.5		

## References

[bb1] Clark, R. C. & Reid, J. S. (1995). *Acta Cryst.* A**51**, 887–897.

[bb2] March, J. (1985). *Advanced Organic Chemistry*, 3rd ed. New York: John Wiley and Sons.

[bb3] Matsumoto, K., Suzuki, M., Iwasaki, T. & Miyoshi, M. (1973). *J. Org. Chem.* **38**, 2731. 10.1021/jo00960a028 4780827

[bb4] Oxford Diffraction (2009). *CrysAlis PRO* Oxford Diffraction Ltd, Yarnton, England.

[bb5] Sheldrick, G. M. (2008). *Acta Cryst.* A**64**, 112–122. 10.1107/S0108767307043930 18156677

[bb6] Shiosaki, K., Fels, G. & Rapoport, H. (1981). *J. Org. Chem.* **46**, 3230–3234.

[bb7] Singh, P. & Totton, E. L. (1981). *Cryst. Struct. Commun.* **10**, 739–743.

[bb8] Totton, E. L., Camp, N. C., Cooper, G. M., Haywood, B. D. & Lewis, D. P. (1967). *J. Org. Chem.* **32**, 2033–2034.

[bb9] Totton, E. L., Freeman, R. C., Powell, H. & Yarboro, T. L. (1961). *J. Org. Chem.* **26**, 343–346.

[bb10] Totton, E. L., Kilpatri, G. R., Horton, N. & Blakeney, S. A. (1965). *J. Org. Chem.* **30**, 1647–1648.

[bb11] Weidlich, H. A. (1938). *Berichte*, **71**, 1601–1603.

[bb12] Westrip, S. P. (2010). *J. Appl. Cryst.* **43** Submitted.

